# Hyperbaric Oxygen Treatment for Carbon Monoxide Poisoning in Italy: Retrospective Validation of a Data Collection Tool for the Italian Registry of Carbon Monoxide Poisonings (IRCOP)

**DOI:** 10.3390/ijerph17020574

**Published:** 2020-01-16

**Authors:** Lidio Maffi, Matteo Paganini, Giuliano Vezzani, Antonios Soumelis, ASPATI Research Group, Enrico M Camporesi, Gerardo Bosco

**Affiliations:** 1 Italian Association of Patients Treated with Hyperbaric Oxygen (ASPATI), 40036 Fidenza, Italy; l.maffi@otip-piemonte.it (L.M.); giuliano.vezzani@alice.it (G.V.); Enrico_Camporesi@teamhealth.com (E.M.C.); gerardo.bosco@unipd.it (G.B.); 2 OssigenoTerapia Iperbarica Piemontese (OTIP), 10135 Torino, Italy; soumelis@gmail.com; 3 Master in Diving and Hyperbaric Medicine, Department of Biomedical Sciences, University of Padova, 35122 Padova, Italy; 4 TEAM Health Research Institute, Tampa General Hospital, Tampa, FL 33606, USA

**Keywords:** carbon monoxide, carbon monoxide poisoning, hyperbaric oxygen therapy, arterial blood gas analysis

## Abstract

*Background*: Carbon Monoxide (CO) poisoning is a frequent cause of intoxication. However, CO poisoning incidence is globally underreported, as well as its features, especially in Italy. The aim of this study was to investigate such characteristics of CO intoxication and foster the creation of the Italian Registry of Carbon Monoxide Poisonings. *Methods*: A data collection tool was developed and organized in five sections: Patient’s characteristics; CO intoxication modality; emergency medical service and emergency department; hyperbaric facility; outcomes. The tool was validated through a retrospective analysis, including CO intoxicated patients treated in 14 Italian hyperbaric facilities between 2015 and 2016. *Results*: A total of 1383 patients were included. The high completion ratio (85%) of the collection tool suggests its feasibility in practical terms. CO intoxications were mostly accidental (93.64%) and caused by solid fuel (48.59%). There was not a uniform application of hyperbaric oxygen treatment protocols, but most of the patients were adequately treated at least at 2.5 ATA for more than 60 min (44.97%). *Conclusion*: This analysis provided new information that was previously unavailable in this country. Furthermore, this tool proved to be a valid base for future registry aiming to consolidate the body of knowledge about CO intoxications in Italy.

## 1. Introduction

Carbon monoxide (CO) poisoning is increasingly recognized as a hazardous and relatively common cause of intoxication. In the United States, CO is estimated to poison about 50,000 patients per year, with mortality ranging between about 1000 and 2000 people per year [1,2]. A similar burden affects WHO European Member States with 140,490 CO intoxications, an average of 342 hospital admissions/year, and an average mortality of 2.24 per 100,000 population reported between 1980–2008 [3].

To achieve better outcomes, a timely treatment cannot be overemphasized. Unfortunately, CO intoxication is a challenging diagnosis since its clinical manifestations can be subtle, nonspecific, and overlap with several other causes. Besides symptoms, the presence of a situation at risk from case history is crucial to pose the suspect and prompt a proper workup in the emergency department (ED) [4]. CO intoxication is confirmed by an increase in carboxyhemoglobin (CO-Hb) blood levels of more than 3% to 4% in nonsmokers and more than 10% in smokers [5].

After removing the patient from exposure, the mainstay treatment for CO poisoning is oxygen administration [4,6]. High-flows of Normobaric Oxygen (NBO) should be administered by mask or endotracheal intubation to any patient suspected of having CO intoxication since the prehospital arena and until confirmation of CO-Hb levels [7]. In the most severe cases, Hyperbaric Oxygen (HBO) can be delivered through hyperbaric chambers to achieve the highest blood oxygen concentration. Although a Cochrane review has not shown any difference between the use of NBO versus HBO to achieve better neurologic outcomes after CO intoxication [8], the latter is supported by experts’ opinions [4,9] as the current best treatment for CO poisoning and is officially endorsed by several national [10] and international [11,12] societies. However, the features and usefulness of HBO treatment (HBOT) for CO poisoning have not been previously assessed in Italy. At the moment, no network among Italian hyperbaric facilities exists and there is no agreement about HBO protocols to treat this ominous intoxication.

With this study, we aimed to develop and pilot the use of a tool, shared among the Italian hyperbaric facilities, as the basis of a future Italian Registry of Carbon Monoxide Poisonings. Furthermore, we intended to collect data and provide the reader with an overview of CO poisoning treated with HBOT in Italy.

## 2. Materials and Methods

### 2.1. Research Tool

A data collection tool focused on the treatment of CO intoxications with HBO was created through a Delphi method during six workshops from December 2015 to December 2017. Medical directors of Italian hyperbaric facilities were selected for their expertise in the field and were involved in the process, under the supervision of three referees from Italian Societies involved in hyperbaric medicine (SIMSI, ASPATI, SIAARTI). A second panel of experts composed by international researchers in hyperbaric medicine reviewed the instrument content for accuracy and provided appropriate final modifications to ensure the validity of the tool. Besides the collaborating center identification part, this template consisted of 20 items organized in five sections: patient’s characteristics; CO intoxication modality; emergency medical service (EMS) and ED; hyperbaric facility; outcomes. The tool is available in Supplementary S1. Finally, after public announcements during academic congresses, 14 Italian hyperbaric medicine facilities voluntarily participated in the study.

### 2.2. Data Collection Procedures

Data were retrospectively collected in 2019 after formal authorization given by each facility’s board. Data and patient charts from 1 January 2015 to 31 December 2016 were analyzed. Only patients with a diagnosis of CO intoxication, treated in the hyperbaric chamber, were included in the study. Items that were unavailable in more than 80% of charts were classified as missing. Times were deduced where available from patient charts.

The collection tool was filled using a Microsoft Office Word 2010 sheet Version 14.0.70 (Microsoft Corporation, Redmond, WA, USA). Data were coded on a master sheet using a Microsoft Office Excel 2010 spreadsheet Version 14.0.47 (Microsoft Corporation, Redmond, WA, USA).

### 2.3. Data Analysis

Frequencies were used to describe respondents’ features; demographic characteristics of the patients were analyzed descriptively through their distribution frequency in the case of qualitative variables, with mean and standard deviation for quantitative variables (when possible). Statistical analysis was performed using IBM SPSS Version 25.0 (IBM, Armonk, NY, USA).

### 2.4. Final Modifications to the Tool

After the end of the study and data analysis, nine items were added to the tool along with the entire panel of results from blood gas analysis to provide more detailed information regarding other features of CO poisoning treatment. These items are marked in Supplementary S1.

### 2.5. Ethical Aspects

Confidentiality of information was ensured, and no financial incentive to participate in the study was offered to the facilities. Since all data were collected such that individual subjects could not be identified or exposed to risks or liabilities, the evaluation was deemed exempt from institutional review approval by the local Ethics Committee (HEC-DSB/04-19).

## 3. Results

A total of 1687 patients were eligible for the study. Of these, 304 were excluded due to unavailable charts, and finally, 1383 were included in the analysis. The completion rate of the tool was 85% (17 filled items out of 20). Data regarding single/repeated exposures and other arterial blood gas values—such as lactates and CO-Hb before the HBO session—were inconsistently reported, so were classified as missing items.

As shown in Table 1, gender was equally distributed. The sample mostly included patients who were more than 12 years old and about half of the individuals were Italian citizens. Solid fuels generated CO intoxication in 48.59% of cases. Of note, 66 patients reported occupational exposure to CO, while 22 were attempted suicides.

The mean CO-Hb value detected in the EDs was 20.1% (±9.1%). CO-Hb mean levels measured at the arrival at hyperbaric facilities were lower (9.1% ± 6.5%), but were only performed in 16.6% of the patients. CO-Hb levels after HBOT dropped to 1.2% (±1.3%) but were detected in just over half the sample (56.9%) (Table 2).

The mean time of exposure to CO was 410 min (±350 min) but was not reported in more than two-thirds of included patients. The time of transport registered from the referring sites to the hyperbaric facilities ranged from 15 to 2880 min, with a mean of 220 min (±200 min) (Table 3).

CO poisoned patients were mostly treated at 2.5 atmosphere absolute pressure (ATA) for more than 60 min (Table 4) and 47.88% underwent a single HBO session (Supplementary S2). No deaths were reported inside the chambers and five patients died after the first HBOT. A small percentage of patients developed cardiovascular or neurologic acute complications within 24 h after the end of the HBO treatment (Table 4).

## 4. Discussion

With this study, we developed and retrospectively tested a data collection tool as the basis of the Italian Registry of Carbon Monoxide Poisonings (IRCOP). The high completion ratio of the collection tool suggests its feasibility in practical terms. Information was easily collected from patients’ charts, except for items regarding exposure and blood gases. Nevertheless, we believe that future prospective and continuous data collection could be more accurate. For this reason, we added further details such as patients’ anamnestic risk factors, symptoms, or NBO administration—which is usually poorly reported in charts but is simple to collect prospectively.

From an epidemiological perspective, CO poisoning incidence is globally underreported, as well as its features [3]. This is the first multi-site study to gather information about CO intoxicated patients treated with HBO in Italy. The presented data not only provide details that were previously unknown in this country, but also have important public health implications that can foster improvements in the treatment of such a subtle disease. Unfortunately, this study considered only HBO-treated patients, thus hampering direct comparisons with previous analyses in the same field [1,2,3]. For the sake of argument, main analogies and differences with current epidemiology will be discussed.

In this study, the most frequent source of the HBO-treated CO intoxication was solid fuel (Table 1). Conversely, a previous analysis from the European area found that gases and vapors were mostly involved in overall CO intoxications [3]. This difference has two possible explanations. Since the latter study analyzed the 1980–2008 period, gas/liquid fuel-powered heating devices could have been less safe than those available at the time of our analysis. In the same vein, several authors described a decline in CO poisoning from the exhaust of motor vehicles [2]. It is also worth noting that charcoal, wood, or pellets seem to predispose to worse CO intoxication requiring HBOT. However this relationship needs to be confirmed in the future on a larger sample representing the overall population of CO intoxicated patients in Italy.

In accordance with literature [2,3], CO poisonings were mostly accidental. Similarly, intentional intoxications accounted for a minority of cases (Table 1), as already described by Hampson in the last decades in the US [2].

The time-lapse between the first contact with the CO intoxicated patient and HBOT can be defined as “Time To Chamber” (TTC). This interval is composed of the times needed to transfer the patient from the site of intoxication to the ED, to suspect and diagnose CO poisoning, to transfer the patient to the nearest HBO facility, and to prepare the hyperbaric chamber. Each of these phases, if not promptly conducted, can significantly delay the start of HBOT and therefore affect the prognosis of patients. However, their reduction is still challenging [4] and poorly assessed in literature [13]. So far, there is no validated optimal treatment threshold time and the debate is still ongoing. Thom and coll. suggested a “golden time” of 6 h [14], while Liao and coll. concluded that HBOT should preferably be performed within 22.5 h after CO poisoning [15]. Nevertheless, this retrospective analysis highlighted a mean transfer time of 3 h and 40 min (Table 3), which is well below the thresholds mentioned above.

HBOT exerts its beneficial effects by increasing tissue concentrations of O_2_ and modulating the inflammatory response [16] as it showed to reduce inflammatory mediators in CO poisoned patients [17]. Despite current recommendations and issued national guidelines [10], Table 4 shows that there is not a uniform application of HBOT protocols for CO poisoning throughout Italy. On the other hand, it is encouraging that only a low percentage of patients received sub-optimal treatment (less than 2.5 ATA), while three-quarters of them were adequately treated at 2.5 ATA or 2.8 ATA for more than 60 min (Table 4). Even if there is not a unique protocol currently recommended in literature, further research is needed to establish better recommendations and help clinicians in tailoring HBOT to the various presentations of CO poisoning.

Patient registries are well-known systems to uniformly collect data about a population affected by a disease or exposed to a specific condition [18]. Given the subtle presentation and difficult diagnosis of CO intoxication, establishing an adequate reporting system is currently challenging, but crucial. Hampson and coll. have previously demonstrated the feasibility of a nationwide, online surveillance system for CO intoxications in the US [19]. This experience originated from a collaboration between the Centers for Disease Control and Prevention and the Undersea and Hyperbaric Medical Society and provided useful and new information to both clinicians and stakeholders on 1907 patients between 2008 and 2011 [20]. Similarly, the future creation of the Italian Registry of Carbon Monoxide Poisonings (IRCOP) will have at least three important implications. First, this registry will lead to better standardization of HBOT for CO poisonings through the creation of a national network adhering to guidelines issued at a country level. Second, it will increase the knowledge about CO poisoning features in Italy. Third, thanks to the increase in understanding, quality improvement measures in public health could be implemented to reduce mortality and ameliorate outcomes of these patients.

The generalizability of these results is subject to certain limitations. For instance, the retrospective nature of the data collected limits interpretation and more granular analyses. However, this study provides information that was not available before and is now accessible to stakeholders. Another drawback is the lack of a structured and shared follow-up network for the CO poisoned, HBO-treated patient throughout the country. Despite the collaborating sites having agreed since 2015 to discharge patients with follow-up instructions, such as a free-of-charge control at the hyperbaric facility after six weeks or referral to general practitioners, all patients were lost to follow-up (personal communication). Since HBOT demonstrated to be promising in reducing CO delayed neurologic sequelae [9], further data are needed to confirm such a correlation, which will be achieved through a rigorous follow-up framework of this subset of patients.

## 5. Conclusions

The tool that was used in this retrospective study proved to be feasible in collecting data about CO poisonings in Italy. With such new information, which was previously unavailable in this country, several quality improvement initiatives will be promoted to ameliorate the outcomes of poisoned patients.

Moreover, this instrument will be used as the framework of the Italian Registry of Carbon Monoxide Poisonings (IRCOP), a new registry aiming to consolidate the body of knowledge about CO intoxications in Italy with several public health implications.

## Figures and Tables

**Table 1:** Demographics of the included patients.

**Patients’ Characteristics**	
**Gender (*n*%)**	
Male	701 (50.69%)
**Age (*n*%)**	
<6 years-old	82 (5.93%)
6–12 years-old	120 (8.68%)
>12 years-old	1176 (85.03%)
Unknown age	5 (0.36%)
**Nationality**	38 countries; 51.27% from Italy. (see Supplementary S2 for details).
**Pregnancy**	0
**CO Intoxication Modality**	
**Source of Intoxication**	
Solid fuel (charcoal; wood; pellets; other)	672 (48.59%)
Liquid/gas fuel (gasoline; diesel; kerosene; methane; propane; other)	490 (35.43%)
Fire and other sources	118 (8.53%)
Missing	103 (7.45%)
**Exposure Modality**	
Accidental	1295 (93.64%)
Work-related exposure	66 (4.77%)
Suicide attempt	22 (1.59%)
**Emergency Medical Service and Emergency Department**	
**Level of Consciousness at presentation**	
Alert	1276 (92.26%)
Depressed level of consciousness with spontaneous breathing	81 (5.86%)
Intubated patient	26 (1.88%)

Sex was equally distributed and most of the patients were more than 12 years old. About half of the patients were Italian citizens. Solid fuels generated CO intoxication in 48.59% of cases.

**Table 2:** **C**arboxy-hemoglobin (CO-Hb) levels detected in the included patients.

Blood Gas Analysis Parameters	Min	Max	Mean	SD	Missing Data [*n* (%)]
CO-Hb level at the EDs (%)	0.0	48.4	20.1	9.1	40 (2.89%)
CO-Hb level before HBOT (%) *	0.0	33.5	9.1	6.5	1154 (83.44%)
CO-Hb after HBOT (%)	0.0	11.0	1.2	1.3	787 (56.91%)
Lactic acid at the EDs (mmol/L)	0.3	29.0	2.1	2.2	532 (38.47%)

SD: Standard deviation; ED: Emergency department; HBOT: Hyperbaric oxygen treatment. Of note, four patients had a CO-Hb value below 1% but were equally treated because of two elements: Symptoms consistent with CO intoxication and high levels of CO detected by EMS crews on the rescue site. Blood gas analysis was not available immediately before and after HBOT, probably because blood gas analyzers were lacking at the hyperbaric facilities. * This item was considered as missing because it was not available in 83.44% of cases.

**Table 3:** Time of exposure to carbon monoxide (CO) and times of transport from the referring sites to the hyperbaric facilities.

Time	Min	Max	Mean	SD	Missing Data [*n* (%)]
CO exposure time	15 min	1440 min	410 min	350 min	964 (69.70%)
Time of transfer from referring sites to the hyperbaric facilities	15 min	2880 min	220 min	200 min	391 (28.27%)

SD: Standard deviation. Data regarding CO exposure were not available in about 70% of the charts. Transport times showed to be highly variable, but mostly below the current literature-recommended threshold. In a few extreme cases (maximum time of transfer: 2880 min), considerable distances, along with logistic and technical failures, hampered prompt treatment.

**Table 4:** Hyperbaric oxygen treatment (HBOT) protocols and outcomes of carbon monoxide (CO) poisoned patients.

HBOT Protocol	*n* (%)
<2.5 ATA (any duration)	55 (3.98%)
2.5 ATA for 60 min	69 (5.00%)
2.5 ATA for more than60 min	622 (44.97%)
2.5 ATA + phase at 2.8 ATA	235 (17.00%)
2.8 ATA for more than 60 min	194 (14.02%)
Other protocols	14 (1.01%)
Missing data	194 (14.02%)
**Deceased during HBOT**	0
**Deceased after the first session of HBOT**	5 (0.36%)
**Acute Complications**	
Cardiac	132 (9.54%)
Vascular	4 (0.29%)
Suspect acute encephalopathy	12 (0.87%)
Pulmonary	0

ATA: Atmosphere absolute pressure. Complications were considered acute if occurring within 24 h after the end of the HBO treatment.

## Supplementary Materials

The following are available online at https://www.mdpi.com/1660-4601/17/2/574/s1, Supplementary S1: Data collection tool. Supplementary S2: Country of origin of patients and number of HBOTs performed. Supplementary S3: Data collected.
