# Supplementary material for: Hyperbaric Oxygen Treatment for Carbon Monoxide Poisoning in Italy: Retrospective Validation of a Data Collection Tool for the Italian Registry of Carbon Monoxide Poisonings (IRCOP)
Source: Int J Environ Res Public Health. 2020 Jan 16;17(2):574. doi: 10.3390/ijerph17020574 (PMC7013710; doi:10.3390/ijerph17020574)
Supplement: Supplementary file 1 [file ijerph-17-00574-s001.zip › ijerph-662829-supplementary PDF/Nov_13_CO ITALY_Supplementary material 1.pdf]

Supplementary Material 1: data collection tool.

**Collaborating Center ID**
**Name, location**
**Date, time**
**1) Patient's characteristics**
**Gender**
**Age (in years; months if < 1-year-old)**
**Nationality**
**Pregnancy (Y/N)** — If YES: Gestation week __________
Anamnestic risk factors: cardiovascular; neurological; metabolic; other (specify)*
**2) CO intoxication modality**
Source of Intoxication (one among these items)
Solid fuel (charcoal; wood; pellets; other)
Liquid/gas fuel (gasoline; diesel; kerosene; methane; propane; other)
Fire
Other
Exposure modality (one among these items)
Accidental (Home; Work-related exposure; other); Intentional
Length of exposure: Minutes ____ OR Unknown
Single exposure OR Repeated exposure
**3) Emergency Medical Service and Emergency Department**
ED Presentation Modality: Self-referral; EMS; referral by a healthcare provider*
Oxygen before HBOT*
Oxygen with mask: during EMS retrieval; at the ED; during ED to chamber transfer.*
Intubation (and where)*
Level of consciousness at presentation (to EMS or at ED)
One among: Alert; Depressed level of consciousness; intubated
GCS (Total and split in Eye-Verbal-Motor)*
Symptoms at presentation (one or more) (to EMS or at ED)*
Neurological (e.g., transitory ischemic attack, seizures)
Cardiovascular (e.g., chest pain, peripheral ischemia)
Other (specify)
Triage Registration Time
First Arterial Blood Gas Analysis (complete)
Time* — pH* — BE* — Lactic acid* — Hb* — Met-Hb* — CO-Hb
**4) Hyperbaric Facility**
Location of the referring site and distance (km)
Time of Transfer = [arrival at chamber time – departure from referring site time]
Time To Chamber (TTC) = [arrival at chamber time – first contact with the patient] *
HBOT protocol
Arterial Blood Gas Analysis before the first HBOT (complete)

Time*	pH*	BE*	Lactic acid	Hb*	Met-Hb*	CO-Hb
**5) Outcomes**						
Arterial Blood Gas Analysis after the first HBOT (complete)						
Time*	pH*	BE*	Lactic acid	Hb*	Met-Hb*	CO-Hb
Deceased during HBOT (Y/N)						
Deceased after the first session of HBOT (Y/N)						
Conditions after the first session of HBOT *						
GCS (Total and split in Eye-Verbal-Motor)*						
Symptoms: asymptomatic; improved; unchanged.*						
Total number of HBOT performed_____						
Troponin values (timing and type of troponin dosed)*						
Acute complications						
Cardiac; Vascular; Neurologic; Pulmonary; Other (specify).						
Chronic complications: Delayed neurologic sequelae; myocardial injuries (Y/N/unknown)*						

ED: Emergency Department; EMS: Emergency Medical Service; HBOT: hyperbaric oxygen treatment; GCS: Glasgow Coma Scale; BE: base excess; Hb: hemoglobin; Met-Hb: methemoglobin; CO-Hb: carboxyhemoglobin; TTC: Time To Chamber.

*These items were not present in the tool developed for the retrospective study but were implemented afterward, to provide further information and more comprehensive analysis in future prospective studies.
