# Supplementary material for: Hyperbaric Oxygen Treatment for Carbon Monoxide Poisoning in Italy: Retrospective Validation of a Data Collection Tool for the Italian Registry of Carbon Monoxide Poisonings (IRCOP)
Source: Int J Environ Res Public Health. 2020 Jan 16;17(2):574. doi: 10.3390/ijerph17020574 (PMC7013710; doi:10.3390/ijerph17020574)
Supplement: Supplementary file 1 [file ijerph-17-00574-s001.zip › ijerph-662829-supplementary PDF/Nov_14_CO ITALY_Supplementary Material 2.pdf]

Supplementary Material 2

Country of origin	n	%
Italy	709	51.27%
Morocco	209	15.11%
Senegal	53	3.83%
Egypt	47	3.40%
Albania	45	3.25%
Romania	27	1.95%
Tunisia	25	1.81%
Pakistan	17	1.23%
India	11	0.80%
Nigeria	11	0.80%
Côte d'Ivoire	9	0.65%
Peru	9	0.65%
Philippines	9	0.65%
Cameroon	8	0.58%
China	8	0.58%
Ecuador	6	0.43%
Guinea	5	0.36%
Turkey	5	0.36%
Burkina Faso	4	0.29%
Ghana	4	0.29%
Mauritius	4	0.29%
Moldova	4	0.29%
Bangladesh	3	0.22%
Germany	3	0.22%
Poland	3	0.22%
Sri Lanka	3	0.22%
Switzerland	3	0.22%
Algeria	2	0.14%
Santo Domingo	2	0.14%
France	2	0.14%
Syria	2	0.14%
United Kingdom	2	0.14%
Benin	1	0.07%
Brazil	1	0.07%
Cuba	1	0.07%
Sierra Leone	1	0.07%
Ukraine	1	0.07%
Venezuela	1	0.07%
Unknown	123	8.89%
Total	1383	100.00%

Most of the patients were from Italy, followed by subjects from Morocco.

Number of HBOTs	n	%
1	662	47.88%
2	305	22.05%
3	6	0.43%
4	5	0.36%
5	4	0.29%
10	1	0.07%
Missing data	400	28.92%

Most of the patients received one hyperbaric oxygen treatment (HBOT). Unfortunately, in 400 patients, it was not possible to verify the number of treatments performed due to data lacking from charts or patients transferred to other facilities.
