# Supplementary material for: Hyperbaric Oxygen Treatment for Carbon Monoxide Poisoning in Italy: Retrospective Validation of a Data Collection Tool for the Italian Registry of Carbon Monoxide Poisonings (IRCOP)
Source: Int J Environ Res Public Health. 2020 Jan 16;17(2):574. doi: 10.3390/ijerph17020574 (PMC7013710; doi:10.3390/ijerph17020574)
Supplement: Supplementary file 1 [file ijerph-17-00574-s001.zip › ijerph-662829-supplementary PDF/Supplementary 3.pdf]

centro	intox	anno
Calabria	152	2015
Calabria	146	2016
Larino	6	2015
Larino	0	2016
DB	*1383*	
**TOTALE**	**1687**	
